# School Functioning of a Particularly Vulnerable Group: Children and Young People in Residential Child Care

**DOI:** 10.3389/fpsyg.2017.01116

**Published:** 2017-07-04

**Authors:** Carla González-García, Susana Lázaro-Visa, Iriana Santos, Jorge F. del Valle, Amaia Bravo

**Affiliations:** ^1^Department of Psychology, University of OviedoOviedo, Spain; ^2^Department of Education, University of CantabriaSantander, Spain

**Keywords:** residential child care, school functioning, school integration, intellectual disability, unaccompanied migrant children

## Abstract

A large proportion of the children and young people in residential child care in Spain are there as a consequence of abuse and neglect in their birth families. Research has shown that these types of adverse circumstances in childhood are risk factors for emotional and behavioral problems, as well as difficulties in adapting to different contexts. School achievement is related to this and represents one of the most affected areas. Children in residential child care exhibit extremely poor performance and difficulties in school functioning which affects their transition to adulthood and into the labor market. The main aim of this study is to describe the school functioning of a sample of 1,216 children aged between 8 and 18 living in residential child care in Spain. The specific needs of children with intellectual disability and unaccompanied migrant children were also analyzed. Relationships with other variables such as gender, age, mental health needs, and other risk factors were also explored. In order to analyze school functioning in this vulnerable group, the sample was divided into different groups depending on school level and educational needs. In the vast majority of cases, children were in primary or compulsory secondary education (up to age 16), this group included a significant proportion of cases in special education centers. The rest of the sample were in vocational training or post-compulsory secondary school. Results have important implications for the design of socio-educative intervention strategies in both education and child care systems in order to promote better school achievement and better educational qualifications in this vulnerable group.

## Introduction

One of the greatest challenges facing child protection in Spain concerns the academic achievement of children and adolescents in residential care. The role of education as a key factor in the process of social inclusion has been widely recognized in Spain (Susinos et al., 2015; Fernández Enguita, 2016). The complexity of modern society is reflected in the growing demand for qualifications in order to access the labor market, which leads to significant inequality between those who continue their schooling and those who drop out (Jackson, 2010; Ward et al., 2014). Nonetheless, for children and adolescents in care, the priority has been to address family and emotional problems, with school being a secondary difficulty in their lives (Trout et al., 2008; Montserrat et al., 2015). It is only recently that attention has started to be paid to school adaptation as an essential aspect of a child's social inclusion (Jackson and Cameron, 2014), highlighting that the transition to adult life, and especially future integration into the workplace, needs certain basic skills which are acquired during schooling. Ferguson and Wolkow (2012) identified the academic environment as one of the most important elements for present and future integration and well-being of minors in care. Furthermore, they argue that the school environment can represent an opportunity to improve a child's resilience when it is a structured, safe setting with dedicated professionals (Höjer and Johansson, 2013).

Current data suggest that children and adolescents in care are in vulnerable situations when it comes to school functioning (Montserrat et al., 2013b). Although most authors would describe the information available as scarce, they all mention the disadvantages facing children and young people in care (Jackson, 2010; Ferguson and Wolkow, 2012; Montserrat et al., 2013a, 2015; Muela et al., 2013), with worse academic results, lower rates of high-school graduation, and entry into post-compulsory education compared with the general population (Snow, 2009; Ferguson and Wolkow, 2012). As part of the YIPPEE project (Montserrat et al., 2013b, 2015), data have been gathered in various European countries which demonstrate a clear difference between the general population and those in the child welfare system in terms of finishing secondary education. They show that in the UK, 41.2% of adolescents in the child welfare system complete their compulsory secondary education, compared to 90.5% of the general population; in Sweden 38% of young people who had been in care had post-compulsory secondary qualifications compared to 85% of the general population and in Denmark 2.5% had post-compulsory secondary qualifications compared to 37.6%, although in this latter case, the number increases when the followup continues to 30 years old (30.8%), according to the data in Montserrat et al. (2015). The situation in Spain has mainly been studied in Catalonia, which highlighted that 31.7% of 15 year olds in care are in the school year corresponding to their age, as opposed to 69.4% in the general population, and a drop out rate from compulsory education of 30.9% in the participating sample of adolescents.

Most research has focused on general school functioning, with less specific research into achievement in different academic areas. The results have generally shown frequent changes of school, expulsions, behavioral problems at school (Trout et al., 2008; Ferguson and Wolkow, 2012; Muela et al., 2013), and academic difficulties related to problems of motivation, attention, learning or cognition (Muela et al., 2013). Recent summaries have highlighted the high probability of being identified as requiring special educational assistance, increased chances of failing or repeating a year, receiving some kind of disciplinary action (Scherr, 2007; Snow, 2009), and presenting higher rates of mental health problems and maladaptive behavior (Zima et al., 2000; Snow, 2009). Some studies have also found gender differences in some areas of school functioning. In general, boys show more problems in school adjustment than girls (Schiff and Benbenishty, 2006; Attar-Schwartz, 2009) and they receive more disciplinary actions (Montserrat et al., 2013a).

The most recent data in Spain covers 10,030 admissions into residential centers in 2015 (Observatorio de la Infancia, 2017). Most children are in residential care due to being in a vulnerable situation in their family of origin. Research has reiterated the impact of these situations on children's social, emotional, and cognitive development (Bravo and Del Valle, 2001; Lázaro and López, 2010; Sainero et al., 2014; Van Vugt et al., 2014; Witt et al., 2016). But this group's difficulties in the academic arena, forgotten for decades in European child welfare systems (Jackson, 2010), are beginning to feature in the scientific literature (Munro and Stein, 2008; Snow, 2009).

Various studies with children and adolescents in out-of-home care indicate different factors related to the appearance and continuation of difficulties in school. Trout et al. (2008) summarized them in a review of 29 international studies, highlighting changes in home placements. This instability leads to significant breaks in schooling making it difficult to develop social relationships or succeed academically, and more likely for behavioral problems to develop at school. These changes can also lead to difficulties in academic supervision by the social educator and interfere with the communication of positive expectations to the children and adolescents about their possible future schooling (Montserrat et al., 2013a). Placement instability has been highlighted in various research as a fundamental factor that negatively affects the child's well-being (Del Valle et al., 2009; Montserrat et al., 2013b; Ward et al., 2014). The nature of the teaching, and the attitudes of teachers, educators and the adolescents themselves may also be factors which affect the child's academic performance. Jackson (2010) highlighted instability, changes of school, and professionals' low expectations about education as obstacles in the way of reducing the schooling gap of those in care. Leonard and Gudiño (2016) did not find that school stability predicted academic results, but did predict internalizing and externalizing problems. Some research has identified greater rejection by classmates, with those in residential care suffering more rejection and being chosen less by classmates for school activities (Martín et al., 2008). Classmates describe them negatively, for example, as having poor relationships with teachers, being aggressive, or seeking attention, which are characteristics that interfere with academic achievement (Martín et al., 2008). A lack of concordance between child welfare services and the education system has also been identified as a barrier to school progress for these children, with one often blaming the other for the children's poor academic achievement (Ferguson and Wolkow, 2012). These difficulties between systems are apparent in the lack of coordination between social educators and teachers (Ferguson and Wolkow, 2012).

Finally, the conditions children experienced prior to entry into the child welfare system, their early experiences, often related to maladaptive functioning in different areas of development, may be having a profound impact on their academic performance (Snow, 2009; Pecora, 2012). Similarly, studies show how it is much more likely to see special educational needs in children in care than in the general population, (Scherr, 2007; Snow, 2009; Trout et al., 2009).

Particular attention should be paid for to two groups: children and adolescents with intellectual disability (ID) and unaccompanied migrant children (UMC). Both groups are excellent examples of the complexity and variety that exists in the profiles of children and young people in residential care and, consequently, the varied needs that residential care programs must address.

ID in children in residential care is a major problem as the proportion is about five times greater than in the general population (Sullivan and Kuntson, 2000; Scherr, 2007; Slayter, 2016). These children present even more educational needs than the other children in care (Trout et al., 2009). Those authors note that the stressors, similar to those experienced by all children in similar situations (change of school or educational program, new rules, and expectations), can increase their vulnerability and therefore raise the probability of various negative outcomes. Research shows that children with disabilities in residential care have social and attentional difficulties, as well as significant deficits in basic academic areas such as reading and academic knowledge (Trout et al., 2009; Sainero et al., 2013). The combination of these deficits with other risk factors in the children's functioning in residential care place this group in a situation of particular vulnerability.

When it comes to UMC, there is a clear consensus that this relatively new phenomenon is one of the most difficult and complex challenges facing child welfare systems. In Spain, the mass arrival of UMC from North Africa between 2000 and 2008 (numbers fell dramatically during the financial crisis) forced regional governments to open large numbers of residential facilities (Bravo and Santos, 2017) and to create new specialized programs to support the social integration of these young people in terms of education and employment, particularly when they reach adulthood. Very little specific information about their adaptation to the school context can be found in our field. One of the few studies which makes reference to the academic arena indicates the poor motivation these young people feel toward schooling (Auger-Voyer et al., 2014) and suggests that this may result from the inadequacy of their previous academic experience (DARNA UNICEF, 1997; Jiménez, 2003; Quiroga et al., 2010; UNESCO, 2012) and the lack of need for qualifications in order to find work in their countries of origin, as well as from their poor literacy and insufficient skill in the language of their adopted country. All of that represents an enormous barrier to their educational adaptation. Nevertheless, these authors indicate, in agreement with other research (Jackson et al., 2005; Kohli, 2009; Hopkins and Hill, 2010; McCarthy and Marks, 2010), that when this lack of motivation is overcome, these young people demonstrate good school progress. In the case of UMC, this adaptation has a significant effect, both as minors and when reaching their majority. So when they are of school age, adaptation makes it easier to widen their social network and feel less isolated (Wade et al., 2005, 2012), or it may be a normalizing experience which helps them feel safer (Hopkins and Hill, 2010; Kohli, 2011; Wade et al., 2012) and increases their sense of belonging, protecting them against certain psychological problems (Rousseau et al., 2004; Sujoldzic et al., 2006; Kia-Keating and Ellis, 2007; Eide and Hjern, 2013). It also makes future adjustment easier (Jackson and Martin, 1998; Masten and Coastworth, 1998; Wade et al., 2005; Miller and Porter, 2007; Casas et al., 2010; Eide and Hjern, 2013), for example, making it easier to find employment, with all the implications that has for stability and social integration (Arnau-Sabatés and Gilligan, 2015).

The data presented in this research is on a topic of widely recognized importance that has been little explored in our context. The study sample from many regions in Spain allows us to describe the present situation in terms of school achievement, and school functioning, and also allows us to analyze the factors related to these difficulties.

### Aims of the present study

The general aim of this research is to analyse school functioning of children in care, in terms of academic achievement, and adaptation to this context. This aim is divided in three specific ones: (1) to describe risk factors (personal, family, clinical, and care process) that may affect school functioning of children in residential care; (2) to describe specific results in school functioning for two vulnerable groups: children with intellectual disability and UMC; (3) to analyze the individual, clinical, and care process factors that are associated with indicators of adaptation in the school context and academic achievement in the general sample.

## Methods

### Participants

There were 1,216 young people who participated in this study (523 girls and 693 boys aged between 6 and 18 years old) (*M* = 13.4 and *SD* = 2.96) who had spent at least 3 months in one of the homes (*n* = 148) in the child residential care network. Children were fostered in different types of residential care facilities: family children's homes (*n* = 87), autonomy programmes for adolescents (*n* = 30), UMC's homes (*n* = 12), homes for children with disabilities (*n* = 3), and therapeutic residential care for young people with emotional and behavioral problems (*n* = 18). The mean stay in care was 42.7 months (*SD* = 37.6). The sample came from children's homes in Asturias, Cantabria, Extremadura, Murcia, Guipúzcoa, Tenerife, and seven SOS Children's Villages in various parts of Spain. Our sample represents the 10% of the total amount of children aged 6–18 years in residential care in Spain (Observatorio de la Infancia, 2017).

### Instruments

#### Sociodemographic and family information

Sociodemographic and family information in each case was obtained via a questionnaire which gathered sociodemographic information and information about variables related to protection measures child care background (time in care home, changes in care homes, history of breakdown of adoptions and fostering, and reasons for going into care). Information about intellectual disability and the condition of UMC were specificly collected to identify this two specific groups.

#### Mental health needs

Information on the mental health needs in each case was obtained from two sources: (1) information related to emotional and behavioral problems was collected if the child was receiving therapeutic care (psychiatric, psychological, and/or psychopharmacological) (2) The Child Behavioral Checklist (CBCL) (Achenbach and Rescorla, 2001) was used to assess and detect patterns of externalizing or internalizing behavior. The CBCL is made up of 113 items which are each scored between 0 and 2 (0 = not true; 1 = somewhat or sometimes true; 2 = often or very often true). The scores of all the items give eight specific clinical subscales and three broadband scales: internalizing, externalizing, and total. The scores were converted into T scores following international scales from which the following ranges were established: normal (≤ 59), borderline (60 ≤ 63), and clinical (≥64) for the broadband scales of internalization, externalization, and total. For the clinical scales or syndromes, the cutoff points in each range were set at normal ≤ 64, borderline 65 ≤ 69, and clinical ≥70. The CBCL has good guarantees of reliability and validity, with a Cronbach Alpha of 0.92 and test-retest reliability of 0.92 for the broadband scales (Achenbach et al., 2008).

#### School functioning

Information about school functioning came from a number of variables: (1) educational level in each case, codified in terms of educational stage that the young person is in (compulsory education, post-compulsory secondary education, vocational/professional training, or other type of study); (2) information on repetition of school years during their education (yes/no); (3) the existence of any kind of modification to their curriculum (yes/no); (4) attendance at a center for special educational needs (yes/no); (5) evaluation of academic success in terms of numbers of subjects passed or failed (good, average, poor); and (6) the school adaptation assessed by social educators by means of 6 questions organized in a Likert-scale from 1 to 5 (1 = never; 5 = always) about frequency of behaviors. This evaluates aspects related to attitudes toward school and school behavior. The internal consistency of this group of items was good with a Cronbach Alpha of 0.889.

### Procedure

This research had official permission from the public bodies responsible for guardianship of children in care. Its design was approved by the ethics committee of the faculty of psychology at the University of Oviedo and all data was collected in accordance with national Law on Personal Data Protection. Data collection was performed in 2013 thanks to financing from the Spanish Ministry of the Economy and Competitiveness through their national research and development plan (PSI2012-33185). Data was collected via key social educators who had been informed of the aims of the study and who followed a procedure designed to guarantee anonymity and data protection.

### Data analysis

Various statistical tests were used depending on the nature of the variables and the group being analyzed. The *Chi-squared* statistic technique was used for the analysis of categorical variables, and the non-parametric *Kruskal-Wallis H* test for the quantitative variables given the size of the groups being compared, and the fact that they did not comply with the assumption of normality. Pairwise comparisons were performed using the *Mann-Whitney* test. To respond the third aim of the study parametric tests were performed using the *Student T*-test and One-way *ANOVA* for the comparison of means, along with the Pearson correlation to estimate the relationship between variables. Finally, a *Stepwise multiple linear regression* analysis was carried out using age, number of changes to care placement and the eight specific clinical subscales from the CBCL as predictor variables, as they had a linear relationship to the criterion variable- school adaptation. Data analysis was done using the statistics program SPSS v19.0.

## Results

### Factors of vulnerability in children in residential care

Table 1 shows the results, separating two groups from the general sample: children with ID, representing 16.3% of the sample, and UMC (7.6%).

**Table 1:** Differences in individual, family, school and care process factors.

**Variable**	**Total (*N* = 1,216)**	**General sample group (*n* = 925)**	**ID (*n* = 198)**	**UMC (*n* = 93)**
	**% or *M (SD)***	**% or *M (SD)***	**% or *M (SD)***	**% or *M (SD)***
Total	100	76.1	16.3	7.6
**Sex**				
Male	57	52.2	61.6	94.6^*^
Female	43	47.8^*^	38.4	5.4
**Age**	13.45 (2.95)	13.09^*^ (3.0)	13.81(2.59)^*^	16.29(0.97)^*^
6–8	8.1	9.6^*^	4.5	–
9–11	17	19^*^	15.7	–
12–14	30.2	32.2^*^	31.8	6.5
15–17	44.7	39.1	48	93.5^*^
**Mean stay**	42.7 (37.6)	41.3(35.7)^*^	60 (45.7)^*^	21.3(19.1)^*^
**Break-down**	13.4	13.3	14	–
**Number of changes of residential facility**	0.90 (1.0)	0.82 (0.96)^*^	0.98 (1)^*^	1.57(1.3)^*^
**Reason for care**				
Physical neglect	47.7	45.4	58.4^*^	–
Emotional neglect	39.7	38.5	45.4	–
Physical abuse	21.6	20.7	25.4	–
Emotional abuse	28.9	28.3	31.4	–
Sexual abuse	4.9	4.3	8.1^*^	–
**Clinical range**	61.4	61.8	68.4^*^	42^*^
Anxiety-depression	10.8	10.5	14.2	6.8
Withdrawal-depression	16.9	15.5^*^	23.2^*^	17
Somatic complaints	8.7	8.9	8.9	6.8
Social problems	18.1	15.2^*^	36.8^*^	8
Thought problems	10.8	9^*^	21.6^*^	6.8
Attentional problems	18.5	17.4	28.4^*^	9.1^*^
Disruptive behavior	25.1	27.9^*^	13.2^*^	22.7
Aggressive behavior	28.5	29.2	27.4	22.7
Internalazing	30.8	30.5	35.8	23.9
Externalizing	51.3	52.9^*^	50.5	36.4^*^
Total	46.6	46	55.8^*^	33^*^
**Mental health treatment**	48.7	47.1^*^	71.9^*^	15.1^*^
**School Indicators**				
**Educational Level**				
Primary and compulsory school	78.5	82.2	80.3	37
Secondary school (post-compulsory)	1.9	2.4	0.5	–
Vocational training	14.1	11	9.1	56.1
Other type studies	2.9	1.7	7.3	4.4
**Curricular adaptation**	39.8	29.7	84.7	41.1
**Academic Performance**				
Good	11.8	12.3	9.2	11
Medium	20.7	18.2	29.1^*^	28.8
Bad	67.5	69.5^*^	61.7	60.3

* *p ≤ 0.05; on chi square for categorical variables and Kruskal-Wallis or U de Mann-Whitney test for quantitative variables*.

There is a slightly higher proportion of boys in the total sample (57%), although this reflects the greater representation of boys in the two highlighted subgroups (χ^2^ = 64.065, *p* < 0.001): children with ID (61.6%) and UMC are mostly boys (94.6%). The proportions are more equal in the remainder of children in residential care (52.2% boys).

The most numerous age group is 15–17 years old (44.7%), followed by the 12–14 year old group (30.2%). The distribution of ages is significantly different in UMC, as 93.5% of them are over 14 (χ^2^ = 106.84, *p* < 0.001).

The mean stay in residential care is very long, three and a half years (42.7 months), and is significantly higher in children with ID, with a mean of 5 years (60 months). For UMC, the mean stay is less than 2 years, which is consistent with these children's situation, where they begin their migration as adolescents with the aim of staying in residential centers until they reach their majority (Kruskal–Wallis test: *H* = 62.879, *p* < 0.001).

In addition to separation from their family of origin, something shared by all the members of this group, 13.4% of the sample had experienced some kind of breakdown in the care process.

Similarly, looking at the number changes of residential centers as an indicator of stability in the process, there is a mean of 0.9 changes (*SD* = 1.0), a little higher for UMC (*M* = 1.57, *SD* = 1.3) (Kruskal–Wallis test: *H* = 40.469, *p* < 0.001).

Table 1 details the different types of threat leading to the adoption of protection measures, more than one type may be present in each child's case. Physical neglect is most frequent (47.7%), and it is important to note the significantly higher prevalence of this type of neglect in children with ID (58.4%) (χ^2^ = 9.796, *p* = 0.002) and the higher levels of sexual abuse in this subgroup (8.1%) (χ^2^ = 4.004, *p* = 0.045). The group of UMC in not included in this description as their reason for being taken into care is their condition of being unaccompanied children.

One important aspect of vulnerability in these children is the presence of emotional and behavioral problems that are clinical according to the criteria of the CBCL. 61.4% exhibited clinical problems in either the internalizing, externalizing or the overall score of the test. The percentage is significantly higher in children with ID (68.4%) and lower in UMC (42%) (χ^2^ = 17.918, *p* < 0.001). The CBCL indicated that more than half of the children presented clinical externalizing problems (51.3%), and 30.8% presented internalizing problems. In the children with ID, significantly more problems were detected in almost all of the internalizing scales: withdrawal (χ^2^ = 6.566, *p* = 0.038), social problems (χ^2^ = 56.307, *p* = 0.000), thought problems (χ^2^ = 27.415, *p* < 0.001), and attentional problems (χ^2^ = 18.278, *p* < 0.001). On the contrary, disruptive behaviors were less frequent (χ^2^ = 18.436, *p* < 0.001).

Almost half of the children were seeing a psychological therapist or psychiatrist (48.7%), with significant differences between the subgroups. 71.9% of children with ID were receiving treatment compared to only 15.1% of UMC (χ^2^ = 85.445, *p* < 0.001).

### Indicators of adaptation and academic achievement

Table 1 also describes the educational situation in the sample using a variety of indicators.

The majority of the general sample (82.2%) and the subgroup with ID (80.3%) are in primary or compulsory secondary education. However, in the group of UMC, it is more common to be in vocational/professional training (56.1%), followed by compulsory education (37%). This difference persists even when looking only at the subgroup of adolescents aged 16 and over, in which 38.1% of UMC are doing vocational training compared to 3.6% of children that age with ID, and 6.6% of the rest of the sample. Very few of the children in any of the three groups are not in any kind of education (between 2.2 and 2.7%).

There are many cases of adaptation of school curricula in children with ID (84.7%), it is important to note that 41.1% of the UMC and 29.7% of the rest of the children in care have also had some curriculum adaptation. This reflects the difficulties this group has in following the standard educational syllabus. The percentage of children attending a special needs school was 7.5%.

The high percentage of children (60.4%) who have repeated a school year in their education is also notable.

In terms of academic performance, most children and adolescents had poor evaluations of their academic achievement (67.5%). The least common evaluation of their performance was “good” (11.8%). (Table 1).

Table 2 details the means and standard deviations for each group in each of the items of adaptation to school, along with the probability associated with the Kruskal-Wallis test statistic. The scores for the children in each of the groups are significantly different. The scores for children with intellectual disability stand out in the “do homework,” “regular attendance,” and “enjoy going” items, whereas in the case of migrant children the items which stand out are “motivation to learn,” “respectful toward teachers,” “good behavior,” and “enjoy going.”

**Table 2:** Differences in school adaptation variables.

**Variable**	**General sample group (*n* = 925)**	**Intellectual disabilility (*n* = 197)**	**UMC (*n* = 92)**	**Kruskal-Wallis *H*-test**	
	***M (SD)***	***M (SD)***	***M (SD)***	**χ2**	***p***
Do homework (daily)^a^^,^ ^b^	3.58 (1.3)	3.68 (1.4)	3.25 (1.4)	6.265	0.044
Motivation to learn^a^^,^ ^c^	3.07 (1.3)	3.02 (1.4)	3.58 (1.2)	13.117	0.001
Regular attendance at school^a^^,^ ^b^^,^ ^c^	4.58 (1.0)	4.76 (0.8)	4.23 (1.2)	27.312	0.000
Respectful toward teachers^c^	3.82 (1.1)	3.86 (1.2)	4.12 (1.3)	6.571	0.037
Good behavior at school^a^^,^ ^c^	3.72 (1.2)	3.7 (1.16)	4.08 (1.1)	9.208	0.010
Enjoy going to school^b^^,^ ^c^	3.52 (1.3)	3.8 (1.19)	3.85 (1.3)	10.728	0.005
Total	22.74 (5.74)	23.06 (6.13)	22.30 (5.79)	2.916	0.233

a *Differences between ID and UMC*.

b *Differences between ID and general sample group*.

c *Differences between UMC and general sample group*.

In almost all of the items, the mean scores range between 3 and 4, although the high score in the three groups in “regular attendance” (4.23, 4.76) is notable. The indicator “motivation to learn” has the lowest mean scores, especially in the ID group and in the remaining sample.

### Factors associated with adaptation to school and academic achievement

Having described school functioning by looking at the differences between the three groups, next, we detail the factors related to school adjustment in the general sample group (*n* = 925) so that the results are not affected by the specific conditions of the comparison groups.

An ANOVA analysis was used to examine the relationship between the main adjustment indicators (adaptation and academic achievement), which was found to be significant and positive. The mean score in school adaptation was 27.93 (*SD* = 2.70) in children with good achievement, 25.15 (*SD* = 3.50) in those with medium achievement, and 20.71 (*SD* = 5.56) in the case of poor achievement (*F* = 121.005, *p* < 0.001). The same result is found looking at each of the six items to assess school adaptation, with the difference being particularly high in motivation to learn and study (*F* = 163.884, *p* < 0.001).

On analyzing the association between the case and clinical variables and academic success, both age and clinical symptomatology as detected by the CBCL demonstrated a significant relationship. The children with poor academic performance were significantly older than those with medium or good performance (*F* = 17.101, *p* < 0.001), with a mean of 13.31 years old (*SD* = 2.69), as opposed to 12.03 (*SD* = 3.39)—12.07 (*SD* = 3.29) in the groups with better academic performance. In terms of the association between clinical symptomatology and performance we found that cases with poor academic performance scored higher in the CBCL subscales of anxiety depression (*F* = 7.361, *p* = 0.001), withdrawal depression (*F* = 6.997, *p* = 0.001), social problems (*F* = 15.838, *p* < 0.001), thought problems (*F* = 11.163, *p* < 0.001), attention problems (*F* = 54.161, *p* < 0.001), rule breaking behavior (*F* = 22.360, *p* < 0.001), and aggressive behavior (*F* = 14.377, *p* < 0.001).

In terms of the relationship with the second criterion variable (adaptation to school), sex, age, number of changes to residential placement, and clinical symptomatology were found to have a significant relationship. Boys had lower scores in almost all of the items (see Table 3). Age was related to worse school adaptation, varying between −0.080 and −0.311 (see Table 4). More changes in placement was associated with lower levels of adaptation in almost all items, although with low levels of correlation (*p* < 0.05, Table 4). All of the CBCL scales had a direct linear negative association with school adaptation, which was especially strong in the *rule-breaking* and *aggressive behavior* scales (Table 4).

**Table 3:** Differences in means in school adaptation by sex.

**Variables**	**Girls**		**Boys**		**Difference in means**	
	***M***	***DT***	***M***	***DT***	***t***	***p***
Do homework	3.72	1.240	3.43	1.328	3.380	0.001
Motivation to learn	3.27	1.288	2.87	1.249	4.690	0.000
Regular attendance	4.55	0.993	4.57	0.990	−0.215	0.829
Respectful toward teachers	4.05	1.075	3.60	1.186	5.959	0.000
Good Behavior at school	4.00	1.096	3.45	1.185	7.137	0.000
Enjoy going to school	3.68	1.262	3.36	1.320	3.675	0.000
Adaptation total	23.28	5.618	21.30	5.794	5.143	0.000

**Table 4:** Correlations between individual factors and school adaptation.

**Variables**	**DH**	**MTL**	**RAS**	**RT**	**GBS**	**EGS**	**AT**
Time in care	−0.038	−0.018	0.017	−0.014	0.010	−0.004	−0.014
N° of placement changes	−0.070^**^	−0.014	−0.081^**^	−0.090^**^	0.050	−0.042	−0.070^**^
Age	−0.311^*^	−0.207^*^	−0.315^*^	−0.118^*^	−0.080^**^	−0.275^*^	−0.272^*^
Anxiety-depression	−0.211^*^	−0.192^*^	−0.138^*^	−0.192^*^	−0.206^*^	−0.168^*^	−0.232^*^
Withdrawal	−0.174^*^	−0.179^*^	−0.137^*^	−0.017	−0.048	−0.168^*^	−0.157^*^
Somatic complaints	−0.159^*^	−0.120^*^	−0.125^*^	−0.138^*^	−0.114^*^	−0.125^*^	−0.167^*^
Social problems	−0.245^*^	−0.278^*^	−0.137^*^	−0.286^*^	−0.302^*^	−0.224^*^	−0.311^*^
Thought problems	−0.260^*^	−0.269^*^	−0.163^*^	−0.263^*^	−0.293^*^	−0.228^*^	−0.314^*^
Attentional problems	−0.404^*^	−0.487^*^	−0.173^*^	−0.371^*^	−0.413^*^	−0.340^*^	−0.463^*^
Rule-breaking	−0.506^*^	−0.488^*^	−0.503^*^	−0.557^*^	−0.569^*^	−0.494^*^	−0.642^*^
Agressive Behavior	−0.397^*^	−0.403^*^	−0.235^*^	−0.551^*^	−0.550^*^	−0.378^*^	−0.526^*^

*DH, do homework; MTL, Motivatión to learn; RAS, Regular attendance at school; RT, Respectful toward teachers; GBS, Good behavior at school; EGS, Enjoy going to school; AT, adaptation total*.

* p < 0.001;

** *p < 0.05*.

A multiple regression analysis was performed to select the variables with the most predictive power over the criterion variable, school adaptation. This analysis was done separately for boys and girls given the modulating effect of the variable sex on the results. The predictor variables introduced were age, number of changes of placement, and the eight specific clinical subscales of the CBCL, given that there was a linear relationship with the criterion variable (see Table 4).

Table 5 gives the standardized coefficients and their probability values for boys and girls. For the girls, model 5 was the most appropriate, explaining 53.5% of the variance (*R*^2^ = 0.535). In this model the rule-breaking variable (β = −0.511) demonstrated most predictive power, although the equation also included problems of attention (β = −0.364), age (β = −0.20), problems of anxiety-depression (β = 0.207), and the number of placement changes (*B* = −0.105). In order to guarantee the model's validity, an analysis was carried out of independence of residuals giving a Durbin-Watson D value of 1.980. For the boys, model 5 also had the most predictive power, explaining 52.5% of the variance (*R*^2^ = 0.525), with rule-breaking again (β = −0.405) having most explanatory power. Other variables included in the equation were problems of attention (β = −0.272), age (β = −0.275), aggressive behavior (β = −0.177), and social problems (β = 0.101). Once again, an analysis of the residuals was done, giving a Durbin-Watson value of 1.821.

**Table 5:** Multiple linear regression analysis for individual and academic variables.

**Regression model**	**B (SE)**	**Beta**	***R^2^***	***t***
**BOYS**				
Rule-breaking behavior	−0.238 (0.029)	−0.405	0.418	−8.313^***^
Attentional problems	−0.155 (0.026)	−0.272	0.458	−6.033^***^
Age	−0.513 (0.066)	−0.275	0.516	−7.728^***^
Aggressive behavior	−0.083 (0.027)	−0.177	0.521	−3.014^**^
Social problems	0.63 (0.030)	0.101	0.525	2.072^*^
**GIRLS**				
Rule-breaking behavior	−0.290 (0.023)	−0.511	0.419	−12.800^***^
Attentional problems	−0.207 (0.024)	−0.364	0.471	−8.797^***^
Age	−0.377 (0.069)	−0.200	0.500	−5.497^***^
Anxiety-depression	0.134 (0.026)	0.207	0.526	5.204^***^
Number of placement changes	−0.612 (0.209)	−0.105	0.535	−2.931^**^

* p < 0.05;

** p < 0.01;

*** *p < 0.001*.

## Discussion

Children and young people in care are a particularly vulnerable group. They have experienced serious adverse family conditions due to neglect or abuse, and they have had to be moved out of home to foster families or residential care, which is another challenge in terms of adaptation and permanency. The consequences of these experiences are well-known, with much research having been done in this area over a long period of time, particularly into behavioral and emotional disorders (González-García et al., 2017). Research into academic and educational development is quite scarce and it is only in the last few years that researchers have begun to pay attention to this topic. This study is an attempt to contribute to the Spanish context, describing some key indicators of academic development from children and young people in residential care.

### Vulnerable factors

The sample description exhibited some of this group's well-known characteristics: residential care in Spain is becoming a specialized program for adolescents (about 45% are over 15), many have had long stays in children's homes (42.7 months on average), which is a matter of some concern in Spain (López and Del Valle, 2015). However, this variable is very different in our two special groups, for young people with ID the average stay increased to 60 months (probably due to the difficulty of placing these children in foster care because of their special needs), but for UMC the average is less than 24 months, as they usually arrive in Spain in late adolescence.

Results related to mental health and well-being showed the high percentage of children diagnosed with ID (16%). This group presents specific needs which are very difficult to meet in heterogeneous peer groups in residential care (Sainero et al., 2013). The results showed that they are the most frequent victims of the more active forms of maltreatment such as physical or sexual abuse, and that they present more emotional and behavioral problems than others in residential care (Casey et al., 2008; Tarren-Sweeney, 2008; Trout et al., 2009). Despite that, this group is almost invisible in international research (Trout et al., 2009).

The data also allow us to confirm the elevated presence of emotional and behavioral disorders, which affect more than 60% of the sample. This high prevalence of problems confirms findings from other countries (Burns et al., 2004; Ford et al., 2007; Bronsard et al., 2011). UMC present fewer disorders, probably because their reasons for being in residential care are not linked to experiences of maltreatment, but rather the desire to migrate to a country with more opportunities (Bravo and Santos, 2017).

### School functioning

Results concerning the subject's educational situation show that about 80% of the children and young people are in compulsory education (which in Spain is up to 16 years old). Only one third of the unaccompanied immigrant minors, however, are in compulsory education, due to the difficulties of the language and their previous low level of education in their birth countries (Jiménez, 2003; Quiroga et al., 2010).

One of the most significant results from the subject's educational situation is the need for curriculum adaptation. About 85% of the children with ID required an adapted curriculum, along with 41% of immigrant minors, but what is particularly remarkable is that 30% of the remainder of the children in the sample also needed curriculum adaptation. The average need for curriculum adaptation in compulsory education in Spain is 5.1% (Ministerio de Educación, Cultura y Deporte, 2016). So children in residential care without ID are six times more likely to need an adapted curriculum at school for other behavioral or developmental reasons.

Another indicator of academic development was repeating one or more school years, which was the case for about 60% of our sample, being the percentage for primary and secondary school in Spain from 15 to 36% according to Ministry of Education data (Ministerio de Educación, Cultura y Deporte, 2016). International research states that children in care repeat course at least in a double proportion than their classmates (Ferguson and Wolkow, 2012) Furthermore, when children's academic achievement is evaluated by residential care workers, almost 68% fall into the “bad” category (meaning that they usually get bad grades in several subjects), and only 11% are considered “good” (they pass all their subjects).

In the school adaptation assessment, results showed that despite the difficulties in academic achievement, the children with intellectual disability scored significantly higher in aspects related to fulfilling certain obligations such as attendance, and doing homework, and exhibit more enjoyment going to school, something which is very important for these children's socialization. UMC showed a higher level of motivation to learn and fewer behavioral problems in general, confirming the aforementioned importance for them to adapt to a new culture and obtain a qualification to start working as soon as possible and begin to look after themselves, similar conclusions that ones pointed out in others researches about unaccompanied children (Wallin and Ahlström, 2005).

### Factors associated to school adaptation and academic achievement

Lastly we looked at the relationship of individual variables (age and sex), case variables (time in residential care, number of placement changes), and clinical variables to the main indicators of school functioning (achievement and school adaptation). In this analysis, both those children with ID and UMC were excluded, given the specific characteristics of those subgroups. The results from the remainder of the sample demonstrated that academic achievement is lower the older the children are, and in those who present clinical symptomatology, whether internalizing or externalizing, something which has been noted in previous research (Cheung et al., 2012).

Worse school adaptation was related not only to being older, but also to sex (girls exhibited better adaptation), and to better scores in the CBCL clinical scales. This was particularly significant with the externalizing scales of disruptive behavior and rule breaking behavior, as well as the attentional problems scale. The presence of clinical problems, especially those which could result in violent, aggressive, or transgressive behavior present a true challenge in the educational environment, as it makes adaptation and adjustment to this social context more difficult, and also affects achievement. We know from previous research (Garland et al., 2001; Burns et al., 2004; McMillen et al., 2005; Jozefiak et al., 2016; González-García et al., 2017) that between 40 and 88% of children in residential care exhibit this type of disorder, which has clear repercussions on school adaptation. Promoting intervention in emotional and behavioral disorders in this population has been identified in previous research as fundamental to improving the process of transition to adult life (Del Valle et al., 2011), a process in which successful adjustment to the educational environment is also key.

One variable which also appeared to be related to worse school adaptation is the number of changes of residential placement. The importance of stability and permanence in out-of-home care, be it family foster care or residential care, has been demonstrated in many studies, and is one of the main challenges of welfare systems (Jackson and Cameron, 2012; Pecora, 2012). It is important to find residential placements which can meet the needs of high demanding children and young people in order to avoid them having to go from one placement to another (Sinclair et al., 2007; Pecora, 2012). Those changes would have a negative impact on academic achievement as they normally involve changes of school and problems of social integration.

The regression analysis carried out to test the predictive value of the variables in the study on the variable adaptation to school environment (carried out separately for boys and girls, given the modulating effect of the gender variable) confirmed the importance of rule breaking behavior, attentional problems, and increased age as the most significant predictors of worse adjustment to the school environment in both boys and girls. The three factors were also associated with worse academic achievement. The impact of externalizing disorders on academic achievement was also found by Harder et al. (2014) with a sample of young people in juvenile justice centers. In boys, the presence of aggressive behavior can be added as a predictor of worse adjustment, whereas the presence of social problems (defined in the scale as children with more infantile behavior and with problems relating to their peers) is associated with better adaptation. In the girls there was something similar, with a higher number of changes to residential placement being an additional predictor of worse adjustment, whereas the presence of anxiety-depression problems was associated with better results for adaptation. It must be remembered that the adaptation variable includes factors such as class attendance, following rules, enjoying going to school, and general motivation. The presence of problems of a more internalizing nature may cause an apparent better adjustment by also being associated with less disruptive behavior. In short, the results seem to indicate that everything which encourages disruptive or transgressive behavior, including, for developmental reasons, getting older, makes it more difficult to adapt to the environment and usually goes hand in hand with worse achievement. Conversely, those factors which inhibit disruptive behavior apparently improve adjustment, but not academic achievement.

In conclusion, the negative impact of disruptive behavior on school adjustment is clear, and its high prevalence in this group, for all the risk factors which these children have in their life histories, has been confirmed. Nonetheless, it is important to note that social problems of an internalizing nature can also impede school functioning, not in such a visible way, but when it comes to evaluating academic progress, with all the impact that may have on these children's futures. Treating these problems, which are very prevalent in this group (Jozefiak et al., 2016), must be established as a priority in intervention.

### Conclusions and recommendations for policy

This study highlights how the educational arena poses one of the challenges in intervention with children and young people in residential care. Efforts should be directed toward improving adaptation and academic achievement owing to their importance in work and social integration once these children and young people have left care. Factors identified as key for academic achievement include stability in the protection measure and in the school, the stable presence of reference adults who are involved, and have expectations of success, along with the involvement of schools in meeting these children's needs (Montserrat et al., 2011). In order for that to happen successfully, the need for coordination between the school and care services is key. Moreover, as this study has shown, there are many whose needs differ from the majority of children and young people in care. UMC and children with intellectual disabilitymust be considered in the design of programs owing to their particular difficulties.

### Limitations and future research

As the results in this study form part of a wider piece of research, it was not possible to look more deeply into the variables involved in school functioning. Future research should collect more variables about educational trayectory of these children and include other key informants such as the children and young people themselves, and teaching staff. In addition, it would be desirable to more deeply examine other school adaptation variables such as relationships with peers. Despite this limitation, the results of this study are enormously important, given the scarcity of research about this topic, as are the implications for policy and practice.

## Author contributions

CG and AB carried out the description of objectives, methodology, results, and discussion. SL and IS focused their contribution on the introduction and researched the state of the art of the topic. JdV carried out main part of discussion. All authors were involved in the original study as researches and reviewed all parts of the manuscript.

### Conflict of interest statement

The authors declare that the research was conducted in the absence of any commercial or financial relationships that could be construed as a potential conflict of interest.
